# Diagnosis Method of Decay-like Composite Insulators in a High-Humidity Environment Based on Characteristic Coefficient of Temperature Rise Gradient

**DOI:** 10.3390/polym15122715

**Published:** 2023-06-17

**Authors:** Yuming Zhang, Sizu Hou, Jianghai Geng, Yijing Gong, Zheng Zhong

**Affiliations:** 1 Hebei Key Laboratory of Power Transmission Equipment Security Defense, North China Electric Power University, Baoding 071003, China; 120222101036@ncepu.edu.cn (Y.Z.); 220212213242@ncepu.edu.cn (Y.G.); 2 Hebei Key Laboratory of Power Internet of Things Technology, North China Electric Power University, Baoding 071003, China; housizu@ncepu.edu.cn; 3 Electric Power Research Institute, China Southern Power Grid, Guangzhou 510663, China; zhongzheng1@csg.cn

**Keywords:** composite insulator, decay-like deterioration, infrared temperature measurement

## Abstract

At present, the temperature rise in insulators is observed using infrared thermometry as a common method of diagnosing decay-like insulators. However, the original characteristic data obtained by infrared thermometry cannot effectively distinguish some of the decay-like insulators from those with ageing sheaths. Therefore, it is imperative to find a new diagnostic characteristic quantity. Based on statistical data, this article first explains that existing diagnostic methods have limited diagnostic effectiveness and a high false detection rate for insulators in a slightly heated state. A full-scale temperature rise test is carried out on a batch of composite insulators returned from the field under high-humidity conditions. Two different defective insulators with similar temperature rise profiles are identified, and an electro-thermal coupling simulation model is developed based on the dielectric characteristic parameters of the above insulators for both core rod defects and sheath ageing. A new infrared diagnostic feature, the temperature rise gradient coefficient, is then obtained to identify the source of abnormal heat in insulators using statistical analysis of an infrared image gallery of abnormally hot composite insulators obtained from field inspections and laboratory tests.

## 1. Introduction

Composite insulators have excellent anti-fouling flash performance, high mechanical strength, easy installation, and are lightweight, and they are widely used in transmission lines during the continuous enhancement of UHV construction in China [1,2]. Since 2012, the use of composite insulators in national grid construction has occupied more than half of the new insulator market, becoming the most used insulator among all types of insulators [3]. The large amount of use means that the performance of composite insulators can have a serious influence on the safe operation of power transmission lines. The long-term mechanical, electrical, and environmental stresses on composite insulators inevitably lead to different degrees of ageing as their service life increases, and the development of internal defects in some composite insulators leads to the occurrence of broken insulator strings [4].

Therefore, how to effectively discern composite insulators with decay-like defects during daily inspections and to take corresponding preventive measures as early as possible to avoid the occurrence of decay-like fracture accidents from the source is an important issue that needs to be addressed for transmission line fault prevention.

The deterioration of decay-like insulators generally starts at the interface between the silicone rubber sheath and FRP mandrel at the high-voltage end and then progresses along the mandrel–sheath interface to the inner mandrel and low-voltage end, with the main features including a crispy and chalky mandrel shaped like dead wood, a chalky and cracked inner surface of the sheath [5,6], etc. In summary, the decay-like defects are hidden defects, and the appearance of decay-like insulators is not significantly different from normal insulators when the sheath is intact, so the decay-like insulators cannot be effectively identified by appearance inspection alone.

In recent years, infrared temperature measurement technology has been applied to diagnose the deterioration of decay-like insulators. Reference [7] reports an analysis regarding an abnormal temperature rise in decay-like fractured insulators before fracture that can be detected by comparing the defect characteristics of in-service abnormally heated insulators with those of decay-like fractured insulators, so infrared temperature measurement technology can be an effective means to diagnose decay-like composite insulators. Reference [8] concluded that the insulator temperature rise within 2K is not considered abnormal heat generation by on-site infrared temperature measurement and simulated pressurization tests of composite insulator temperature rise. Reference [9] classified the temperature rise defects of composite insulators into five classes based on their temperature rise states and temperature rise types and proposed the O&M maintenance strategy accordingly. Reference [10] conducted temperature rise tests on natural decay-like and artificially simulated defective insulators under laboratory environmental conditions and proposed the characteristics of the length of the heating area, the location of the heating, and the uniformity of temperature distribution of the compound insulator heating defects as the criteria for judging the causes of abnormal heating of compound insulators. Reference [11] studied and analysed the abnormal heating fault of the composite insulator in field operation, and divided the abnormal heating of composite insulators into two types: point temperature rise and segment temperature rise, and pointed out that the segment temperature rise has a larger amplitude and wider temperature rise range than the point temperature rise; in addition, the point temperature rise phenomenon is only effective in a high-humidity environment, while the segment temperature rise insulator also produces a large temperature rise in a low-humidity environment. At present, there are two main types of reasons known to cause an abnormal temperature rise in composite insulators. One is caused by external insulation factors, such as ageing and moisture absorption of the sheath [12], pollution [13], improper configuration of the grading ring leading to end field strength distortion [14], etc.; the other is caused by internal insulation factors, such as mandrel defects [15], core rod sheath interface defects [16], etc. The mechanism of abnormal heat generation in composite insulators has not yet formed a systematic and perfect theory, and the temperature rise pattern caused by different heat sources (internal insulation factors represented by mandrel defects and external insulation factors represented by sheath ageing) is not clear.

Most of the existing infrared detection methods identify the heat source of abnormally heated insulators using two types of temperature rise characteristics: the degree of temperature rise and the characteristics of the temperature rise area, and they can effectively diagnose composite insulators with severe heat generation. However, when the degradation of composite insulators is at a low degree, the heat generated by the polarization loss can be lost quickly through heat conduction, resulting in an insignificant temperature rise, which is difficult to be diagnosed and detected using infrared temperature measurement technology. In this case, the abnormal temperature rise at the high-voltage end can only be observed when moisture intrudes into the degraded mandrel under high-humidity conditions, thus intensifying its polarization loss and generating heat which then accumulates in the sheath [17,18]. However, the temperature rise characteristics of the decay-like mandrel at this time are very similar to those caused by moisture absorption due to sheath ageing [19,20,21], and the application of the traditional temperature rise characteristic quantity has limited diagnostic effect and a high false detection rate for such insulators with similar heat generation. Therefore, a new characteristic quantity needs to be proposed to analyse and diagnose such insulators.

In view of this, this paper first analyses the limitations of the commonly used diagnostic characteristic quantities through the available infrared temperature rise data of composite insulators. A full-scale temperature rise test under high-humidity conditions is carried out on a batch of composite insulators returned from the field. Two insulators with similar temperature rise and different defect types are identified, and their dielectric characteristics are measured. Based on the energy functional variational principle, a finite element simulation model for the abnormal heating of composite insulators at the high-voltage end is established, and the distribution characteristics of the surface temperature rise under the two conditions of mandrel defects and sheath ageing are discussed to provide a basis for the derivation of new characteristic quantities. Based on the simulation results, the infrared images of abnormally hot composite insulators obtained from field inspection and laboratory tests are statistically analysed from the perspective of temperature rise gradient, and finally, a new infrared diagnostic characteristic quantity, the temperature rise gradient coefficient, is obtained which can effectively determine the cause of abnormal heat at the high-voltage end.

## 2. Statistical Analysis of Information on the Temperature Rise Characteristics of Composite Insulators under Different Defect Conditions

Infrared imaging technology is an effective method of diagnosing decay-like composite insulators. The infrared images include two main types of information: the temperature rise region and the magnitude of the temperature rise in the target object. The majority of studies have proposed criteria for the diagnosis of decay-like composite insulators based on these two types of temperature rise information. To verify the validity of the diagnosis of decay-like insulators by temperature rise area and temperature rise amplitude information, this section presents a statistical analysis of the infrared temperature rise images of composite insulators acquired during field and test acquisition based on these two types of temperature rise characteristics.

### 2.1. Diagnosis Method Based on Temperature Rise Area Characteristics

**Figure 1:**
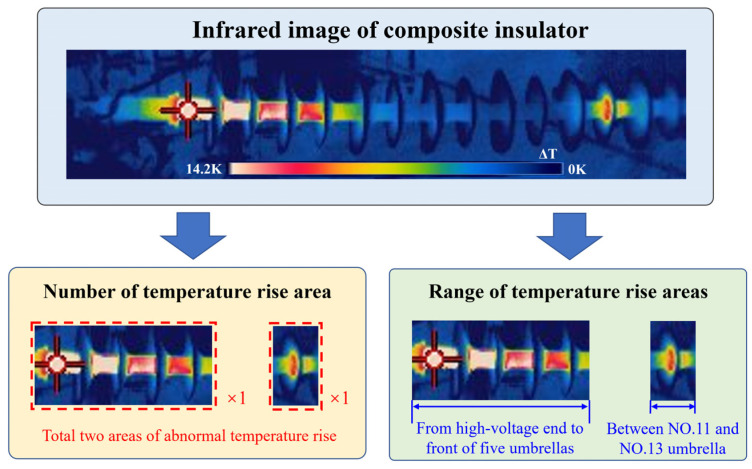
Characteristic information of temperature rise region of abnormally heating composite insulators.

Previous report [11] shows that both the number of temperature rise regions and the range of temperature rise regions can be extracted directly from the infrared image of a composite insulator. A schematic diagram of the extraction of temperature rise region features from an abnormally heated composite insulator is shown in Figure 1.

**Figure 2:**
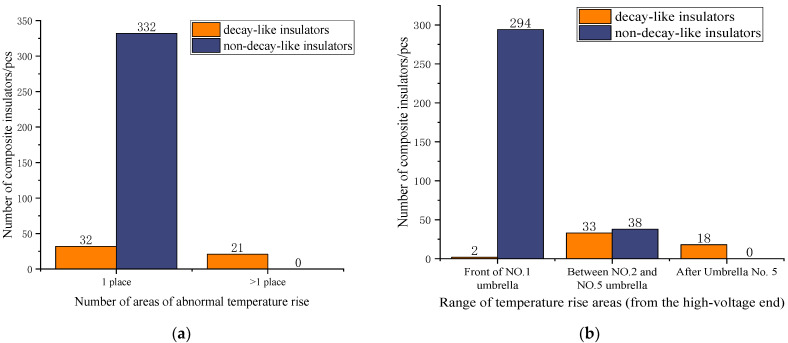
Abnormal heating composite insulator temperature rise area range and quantity statistical information. (**a**) Number of temperature rise areas; (**b**) range of temperature rise areas.

The area with a temperature rise amplitude Δ*T* > 1 °C was defined as the abnormal temperature rise area of composite insulators [22]. The number of temperature rise areas and the range of temperature rise areas in a total of 385 composite insulators, including 53 decay-like composite insulators and 332 non-decay-like composite insulators, obtained from operation sites and tests were statistically calculated, and the statistical results are shown in Figure 2.

By analysing the statistics on the number of temperature rise areas, it can be concluded that a composite insulator can be diagnosed as a fritillary composite insulator when there is more than 1 temperature rise area, but for composite insulators with only 1 temperature rise area, further analysis is required through the temperature rise area range. By analysing the statistical data on the range of temperature rise areas, it can be concluded that if the temperature rise area extends from the high-voltage end fittings to after the 5th umbrella, it can be diagnosed as a decay-like composite insulator; if the temperature rise area is limited to the first umbrella, it can be diagnosed as a non- decay-like composite insulator. For the composite insulators with temperature rise areas between the high-voltage end fittings and the second to the fifth umbrella, the number of non-decay-like insulators and decay-like insulators is comparable, and there is a possibility of misjudgement. Further information on temperature rise characteristics is required for diagnosis.

### 2.2. Diagnosis Method Based on Temperature Rise Amplitude Characteristics

**Figure 3:**
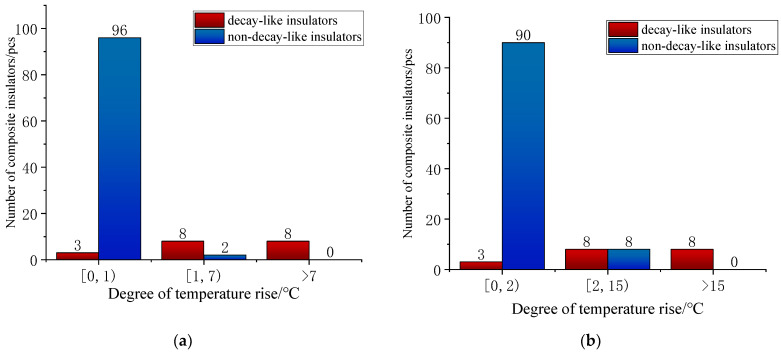
Experimental measurement of temperature rise data in high- and low-humidity environments. (**a**) Low-humidity condition (30%RH); (**b**) high-humidity conditions (75%RH).

When a composite insulator has only one abnormally hot area between the high-voltage end fixture and the second to the fifth umbrella, there is no way to effectively identify the decay-like insulators by the characteristics of the temperature rise area, so further judgement is required using the characteristics of the temperature rise amplitude. In this paper, the temperature rise amplitude data for the 117 composite insulators (19 decay-like insulators and 98 sheath-aged insulators) with abnormal heating at the high-voltage end only, under high- and low-humidity conditions, are shown in Figure 3, where the relative humidity is 30% for the low-humidity conditions and 75% for the high-humidity conditions.

According to the data on the temperature rise amplitude of decay-like and non-decay-like composite insulators in high- and low-humidity environments, 98.0% of non-decay-like insulators have a temperature rise amplitude within 1 °C, and 84.2% of decay-like insulators have a temperature rise amplitude greater than 1 °C. Therefore, the abnormal heating of composite insulators with a temperature rise amplitude greater than 1 °C in a low-humidity environment can be diagnosed as a composite insulator caused by mandrel decay. In the high-humidity environment, 91.8% of the non-decay-like insulators and 15.8% of the decay-like insulators have a temperature rise amplitude of less than 2 °C. Therefore, abnormally heated composite insulators at this temperature rise amplitude can be diagnosed as heat due to sheath ageing, etc. Composite insulators with a temperature rise amplitude greater than 15 °C can be diagnosed as abnormally heated due to decay. However, when the temperature rise amplitude is between 2 °C and 15 °C, the number of non-decay-like and decay-like insulators is comparable. In this temperature rise range, misjudgements can be made by using only the temperature rise amplitude as a diagnostic feature.

To sum up, it is not possible to effectively diagnose decay-like insulators with abnormal temperature rise only at the high-voltage end and with temperature rise amplitude between 2 °C and 15 °C through two types of information: temperature rise area and temperature rise amplitude. It is necessary to propose new feature quantities to effectively distinguish the defect types of composite insulators with similar heating conditions.

## 3. Infrared Inspection Test of a Batch of Returned Insulators

**Figure 4:**
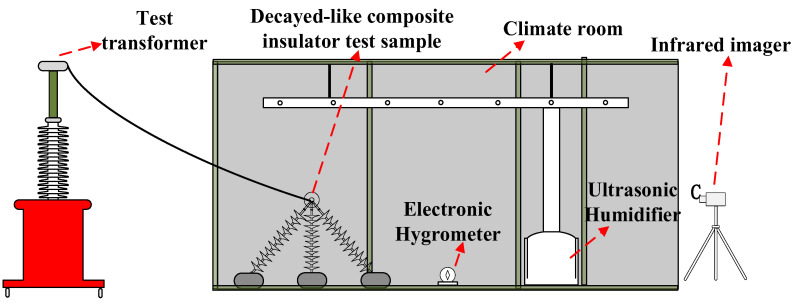
Diagram of the test arrangement.

A full-scale temperature rise experiment was carried out on a batch of 26 field retired 500 kV insulators in a high-humidity environment (RH = 75%). The full-scale temperature rise experiment was carried out in an artificial climate chamber with a continuously adjustable relative humidity range of 30% to 95% with a tolerance of ±5% and a stable humidity value within 30 min. Due to space constraints, the composite insulator samples were placed horizontally, with the high-voltage end supported by an insulating bracket and the low-voltage end extended outside the artificial climate chamber, with the entire sample suspended 1.3 m above the ground. The arrangement of the experiment is shown in Figure 4.

The temperature rise in the composite insulator was recorded with a FLIR-E60 handheld infrared imager with a resolution of 320 × 240 pixels, a measurement temperature range of −20–120 °C, and a setting of 0.9 IR emissivity.

The test steps are as follows:

(1) Before the test, place the composite insulator samples in the artificial climate room, adjust the humidity in the climate room to 75%, and stabilise for 30 min, to ensure an even distribution of humidity in the climate room.
(2) After the humidity in the climatic chamber is stabilised, a frequency voltage is applied to the composite insulator sample, and the temperature rise in the test article is stabilised after 60 min of voltage application, at which time the temperature rise in the sample is recorded at an observation distance of 7 m, respectively.

One composite insulator with more than one abnormal temperature rise area was found during the test, which was confirmed to have decay in the mandrel after an inspection by dissection. Seven insulators were found to be abnormally hot at the high-voltage end. Three of these insulators had a maximum temperature rise of less than 2 °C, and after an inspection, it was confirmed that the mandrel did not deteriorate and was judged to be abnormally heated due to ageing or fouling of the sheath. Two insulators with a maximum temperature rise greater than 15 °C were dissected and inspected to confirm that the mandrel had deteriorated. In addition, the maximum temperature rise in two returned insulators was between 2 °C and 15 °C.

**Figure 5:**
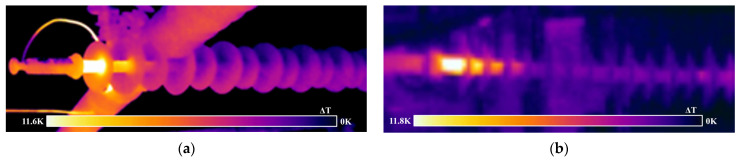
Infrared images of composite insulators with different defect conditions. (**a**) Sheath ageing (S1); (**b**) decay-like (S2).

The test results for the above two insulators are shown in Figure 5. The maximum temperature rise in the decay-like composite insulator was 11.8 °C, with the temperature rise area extending from the high-voltage end fitting to the front of the fourth umbrella, and the maximum temperature rise in the sheathed aged composite insulator was 11.6 °C, with the temperature rise area extending from the high-voltage end fitting to the front of the second to third umbrella. Before dissection and inspection, it is difficult to accurately diagnose whether the two are decayed from the degree of temperature rise and the characteristics of the temperature rise area alone.

**Figure 6:**
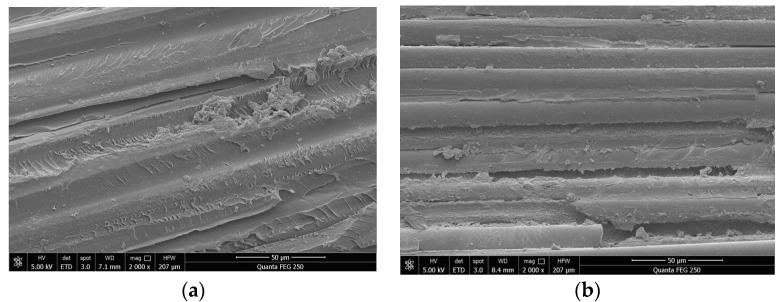
Microscopic appearance of the surface of the mandrel in the heating area of two specimens. (**a**) S1 test sample; (**b**) S2 test sample.

Scanning electron microscopy (SEM) tests were carried out on the core rods of these two insulators, as shown in Figure 6. The epoxy resin matrix on the surface of the core bar of insulator number S2 showed degradation and a large gap between the fibres.

**Figure 7:**
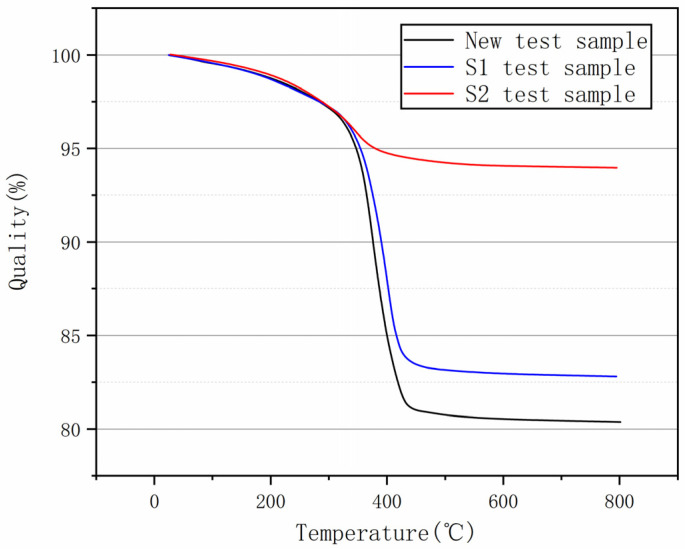
TGA results of different samples.

The results of the thermogravimetric analysis are shown in Figure 7. The quality of the specimen decreased slowly with the rise in temperature before the temperature reached 320 °C. At this stage, the epoxy resin had not yet degraded, and the decrease in quality was caused by moisture. When the temperature reaches 430 °C the core mass reaches its lowest value and does not change with increasing temperature, i.e., there is no longer any epoxy resin in the core, and the remaining mass is glass fibre. The epoxy resin content of the new composite insulator mandrel tested was 19.6%, while the epoxy resin content on the surface of the mandrel in the hot part of insulator number S1 was 17.1%, and the epoxy resin content on the surface of the mandrel of insulator number S2 was 5.9%.

The results of SEM and thermogravimetric analysis showed that the reason for the abnormal heating of the S1 specimen was the decay of the core rod and that the reason for the abnormal heating of the S2 specimen was the ageing of the sheath.

In order to further analyse the causes of abnormal heating of composite insulators and to provide a basis for simulation modelling, the Novo-control broadband dielectric impedance spectrometer produced by Novocontrol Technologies GmbH&Co. KG (Frankfurt, Germany) and the Agilent-4980A tester produced by Agilent Technologies Inc. (City of Santa Clara, CA, USA) were used to measure the parameters of dielectric constant (*ε*) and saturated hygroscopic loss (tan*δ*) for the above two specimens, and the test results are shown in Table 1.

## 4. Simulation Analysis of Abnormal Temperature Rise Characteristics of Composite Insulator High Voltage Ends under Different Defect Conditions

### 4.1. Simulation Model

The 3D model used for the simulation was built using the parameters of the model FXBW-500/300 composite insulator, which are shown in Table 2.

**Figure 8:**
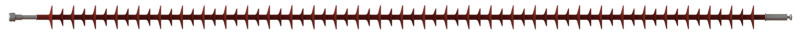
Composite insulator model.

The composite insulator is mainly composed of a glass fibre epoxy resin core rod (GFRP), a silicone rubber sheath (SIR), and a fitting at both ends. The completed 500 kV composite insulator model is shown in Figure 8.

### 4.2. Calculation Methods (Dielectric Losses, Heat Transfer Theory)

**Figure 9:**
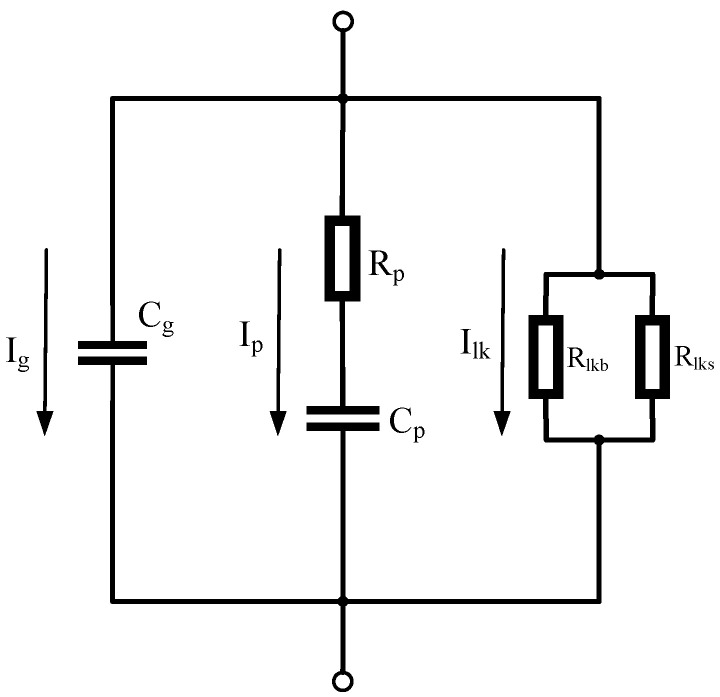
Dielectric equivalent circuit diagram.

The abnormal heating of composite insulators in operation is mainly attributed to the dielectric loss caused by the dielectric polarisation effect under the action of the frequency of the AC electric field and the loss caused by the leakage current [17]. A dielectric equivalent circuit model is shown in Figure 9. In the figure, *C_g_* is the capacitance formed by lossless polarisation; *R_p_* and *C_p_* are the equivalent resistances and capacitances formed by lossy polarisation; *R_lk_* is the leakage resistance, which can be further subdivided into body leakage resistance and surface leakage resistance; and *I_lk_* is the leakage current.

Furthermore, the power loss per unit volume of the dielectric can be expressed as

(1) $$p=EJ_{r}=E^{2}\omega \varepsilon \tan\delta$$

where *E* is the electric field strength distribution of the dielectric, *J_r_* is the total active current density in the dielectric, *ω* is the magnitude of the angular frequency of the sinusoidal electric field, *ε* is the dielectric constant of the dielectric and tan*δ* is the loss angle tangent of the dielectric. This formula shows that the power loss of the dielectric is closely related to the magnitude of the electric field and explains why most of the heated areas are concentrated at the high-voltage end of the abnormally heated insulator.

**Figure 10:**
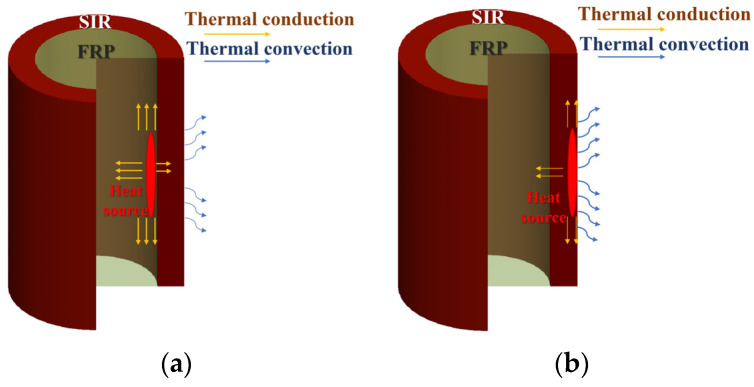
Schematic diagram of the heat transfer process of composite insulator. (**a**) Mandrel deterioration; (**b**) sheathing problems.

Heat conduction, heat convection, and heat radiation are the three basic forms of heat transfer in heat transfer theory. In the case of load balance, the influence of heat radiation from objects is generally not considered [23], therefore only the first two forms of heat transfer are discussed in this paper. The heat generated by the deteriorated mandrel or aged silicone rubber sheath due to dielectric loss is mainly transferred in the form of heat conduction inside the composite insulator and heat transfer through thermal convection on the surface of the composite insulator; the specific process is shown in Figure 10.

When the dielectric is stationary and there is no exchange of power with the outside environment, by the law of conservation of energy, the differential equation for heat conduction can be obtained as follows:

(2) $$\left\{\begin{array}{l} q=-k\nabla T\\ \rho c\frac{\partial T}{\partial t}=-\nabla \cdot q+q_{v} \end{array}\right.$$

where ***T***(*x*,*y*,*z*,*t*) is the spatial and temporal distribution of the temperature field, ***q*** is the heat flow density (W/m^2^), *k* is the thermal conductivity (W/(m·K)), *ρ* is the density of the dielectric (kg/m^3^), *c* is the specific heat capacity of the dielectric (J/(kg·K)), and *q_v_* is the intensity of the heat source (W/m^3^).

On the outer boundary of the field, which is the interface between the sheath of the composite insulator, the end fittings, and the air, convective heat transfer can be expressed as

(3) $$-n\cdot q=h(T_{1}-T)$$

where *h* is the convective heat transfer coefficient in W/(m^2^·K) and *T*_1_ is the external temperature.

After the partial differential Equations (2) and (3) applicable to the analysis of the temperature field of a composite insulator have been obtained, the following Lagrangian generalized integral equation can be obtained using the variational method.

(4) $$J=\int_{\Omega }(\frac{k}{2}{\left\|\nabla T\right\|}^{2}-q_{v}T)d\Omega +\int_{\partial \Omega }\frac{h}{2}{\left(T_{1}-T\right)}^{2}d\Gamma$$

The finite element equations can be obtained by discretising the above general integral equations and solving for the minima, which will not be further described in this paper. The relevant thermal parameters of the composite insulator are shown in Table 3 [24].

### 4.3. Analysis of Temperature Rise Characteristics of Composite Insulators under Different Defect Conditions

The simulation of a 500 kV composite insulator with thermoelectric coupling was carried out using finite element simulation software. The focus of this paper is to investigate the differences in heat generation and heat transfer between different heat source cases when similar temperature rise amplitudes occur at the high-voltage end of the insulator. From Equation (1), the dielectric loss power is related to the loss angle tangent and dielectric constant of the material. In this paper, concerning the relevant dielectric property experimental data of Section 2, the mandrel degradation abnormal temperature rise model and the sheath ageing abnormal temperature rise model with a temperature rise of about 10 °C at the high-voltage end were obtained, respectively, by setting the ageing material parameters at the corresponding positions in the simulation model.

**Figure 11:**
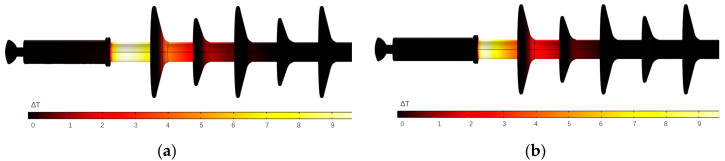
Surface temperature rise distribution of composite insulator. (**a**) Mandrel deterioration; (**b**) sheath ageing.

Assuming an initial temperature of 15 °C and humidity of 90% for both the environment and the composite insulator, the temperature rises on the surface of the composite insulator are distributed as shown in Figure 11 when an industrial frequency voltage is applied to the high-voltage end of the insulator and left until the temperature stabilises.

**Figure 12:**
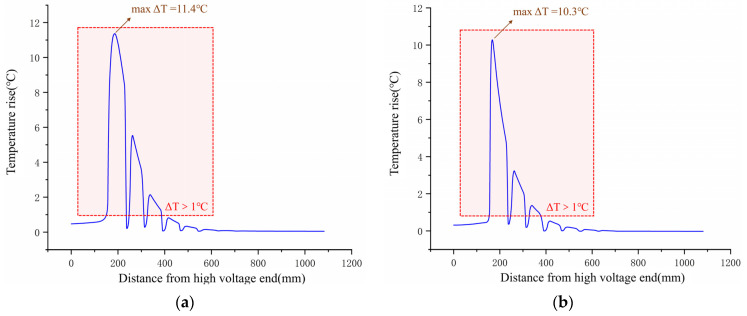
Temperature rise distribution curve of high-voltage end of composite insulator. (**a**) Mandrel deterioration; (**b**) sheath ageing.

It can be initially seen from Figure 9 that, at the high-voltage end of the composite insulator, the temperature rise distribution is more concentrated for the sheath-aged insulator and more evenly distributed for the mandrel-deteriorated insulator. The temperature rise curve on the axis of the high-voltage end of the composite insulator surface has been extracted from the temperature rise distribution image, as shown in Figure 12.

The information on the degree of temperature rise and the characteristics of the temperature rise area read from the temperature rise distribution curve on the axis of the high-voltage end surface of the composite insulator is shown in Table 4.

**Figure 13:**
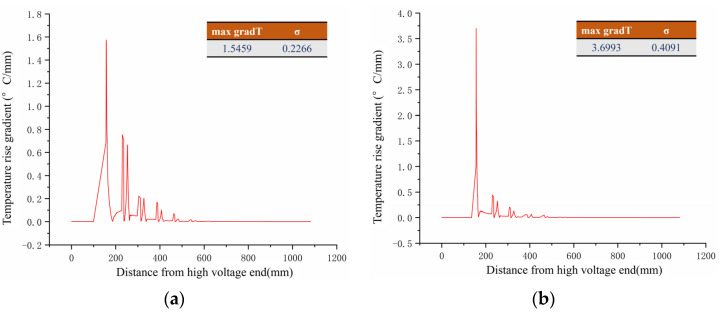
Temperature rise gradient curve of high-voltage end of composite insulator. (**a**) Mandrel deterioration; (**b**) sheath ageing.

It can be seen from Table 3 that the difference in the degree of temperature rise and the characteristics of the temperature rise area between the two defect cases are not significant. The temperature rise gradient curve on the high voltage end axis of the composite insulator is further calculated from the temperature rise distribution curve of the high-voltage end axis, as shown in Figure 13.

Calculating the temperature rise gradient on the axis of the high-voltage end showed that the maximum temperature rise gradient on the surface of the mandrel-deteriorated insulator was 1.546 °C/mm, while the maximum gradT on the surface of the sheath-aged insulator reached 3.699 °C/mm. There was a significant difference in the maximum temperature rise gradient between the two insulators. Furthermore, the average variance σ for the mandrel-degraded insulators was 0.2266, while the average variance σ for the sheath-aged insulators reached 0.4091, indicating that the significant areas of temperature rise gradient for the sheath-aged insulators were more concentrated, while the mandrel-degraded insulators had a more even distribution of temperature rise gradients, which is also the same conclusion drawn from the composite insulator surface temperature rise distribution images.

### 4.4. Analysis of the Causes of the Variability of the Axial Temperature Rise Gradient of Composite Insulators under Different Defect Conditions

To investigate the causes of the differences in temperature rise gradients on the surface of composite insulators for different defective cases, this section further analyses the heat source areas and heat transfer of composite insulators for different defective cases.

**Figure 14:**
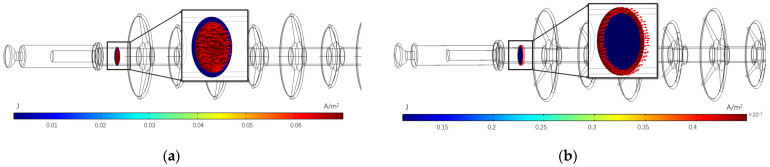
Current density distribution at high-voltage end of composite insulator. (**a**) Mandrel deterioration; (**b**) sheath ageing.

The current density flowing in the dielectric can characterise the magnitude of the dielectric loss power. To investigate the differences in the heat source areas for the two degradation cases, the current density at one cross section of the high-voltage end is calculated as shown in Figure 14.

As can be seen from Figure 14, the distribution of leakage currents in the insulator differs between the two cases. When the mandrel is degraded, the leakage current in the insulator is mainly through the mandrel, with the heat source concentrated in the mandrel. In the case of sheath ageing, the leakage current in the insulator is mainly through the sheath, with the heat source concentrated in the sheath.

**Figure 15:**
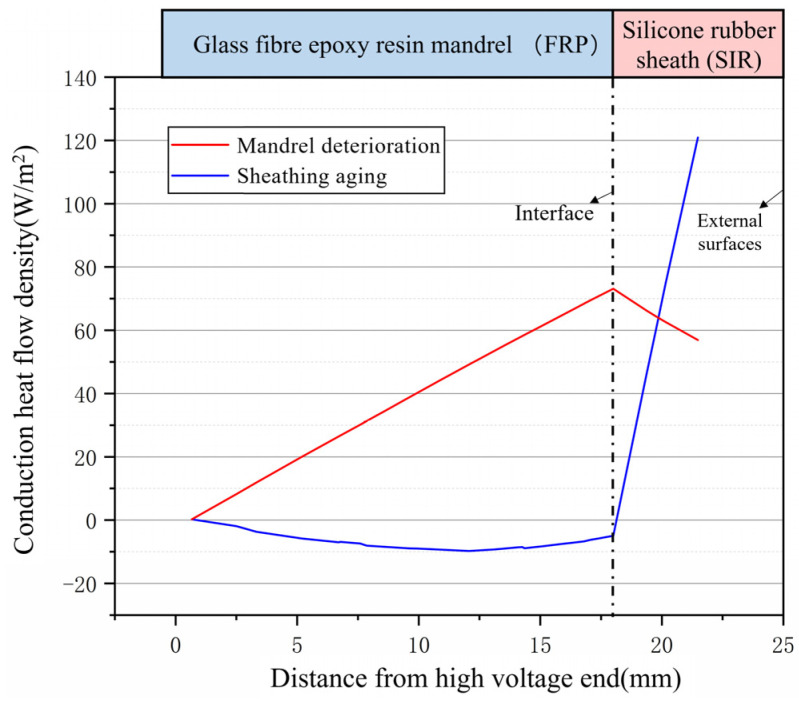
Radial conduction heat flux density distribution at high-voltage end of composite insulator.

The following analysis of the heat transfer characteristics of the composite insulator is carried out for different heat source locations. As shown in Figure 15, the density distribution of radially conducted heat flow at a location of the high-voltage end of the composite insulator is presented in the simulation.

As can be seen from Figure 15, for mandrel-deteriorated insulators, the heat flow density increases due to the continuous accumulation of heat during the inside-out conduction of heat in the mandrel region. As the thermal conductivity of silicone rubber is less than that of FRP, heat transfer is impeded when heat is conducted to the sheath area and the heat flow density is consequently reduced. Due to the fact that the heat source of sheath-aged insulators is concentrated in the outer layer, the outer surface of the sheath is in direct contact with the air, and convective heat transfer is evident, with most of the heat generated by the heat source being transferred directly to the outer air via convection. Therefore, the closer to the sheath surface, the greater the density of heat flow to the outside, while conversely, less heat is transferred from the sheath to the inside.

**Figure 16:**
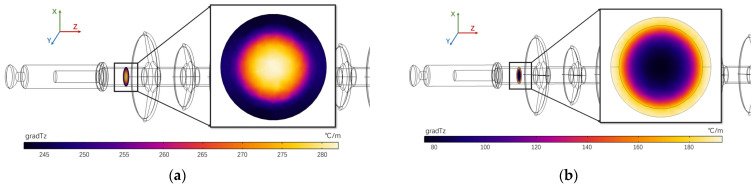

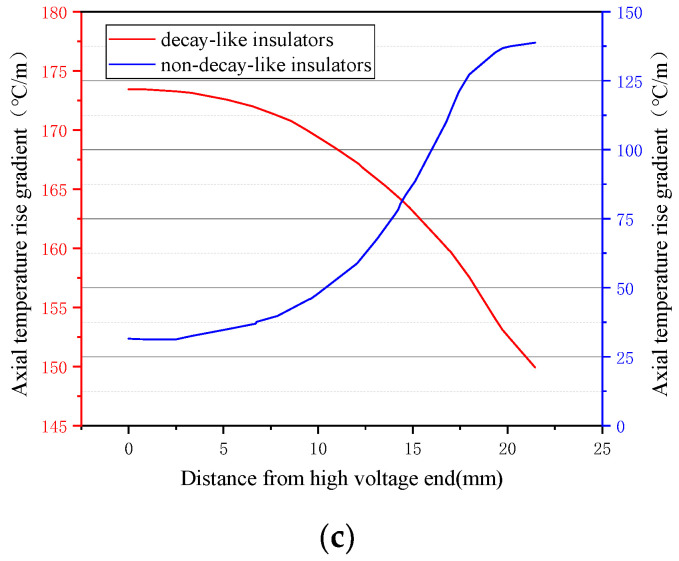
Axial temperature gradient distribution of high-voltage end of composite insulator. (**a**) Mandrel deterioration; (**b**) Sheath ageing; (**c**) axial temperature gradient distribution at the high-voltage end.

Combining the above differences in the location of the heat source and the radial heat transfer of the composite insulator under different defect conditions, a cross section of the high-voltage end of the composite insulator was taken to calculate the magnitude of the axial temperature gradient on its surface, and the results were obtained as shown in Figure 16.

It can easily be seen from Figure 16 that the axial temperature gradient in the mandrel of the degraded composite insulator is greater than the axial temperature gradient in the sheath due to differences in the location of the heat source and radial heat transfer, while the axial temperature gradient in the mandrel of the aged composite insulator is less than the axial temperature gradient in the sheath. In other words, the axial temperature on the surface of the degraded insulator is more evenly distributed compared to the axial temperature distribution of the inner mandrel.

In summary, due to the different locations of the heat sources and the differences in heat transfer characteristics caused by the thermal conductivity of the materials, the temperature gradient distribution of the composite insulators with similar temperature rise amplitudes at the high-voltage end of the insulators with different causes of deterioration can differ significantly, specifically in terms of the maximum value of the temperature rise gradient and the average variance. Therefore, a new characteristic quantity can be constructed based on these two parameters to effectively identify the cause of heat generation.

## 5. Simulation Analysis of Abnormal Temperature Rise Characteristics of Composite Insulator’s High-Voltage Ends under Different Defect Conditions

The temperature distribution curve on the insulator surface axis can be obtained by extracting the grey scale distribution of the abnormally heated composite insulator at the axis position in the infrared image [25,26]. In this paper, based on the commonly used IR diagnostic feature quantity, the temperature rise gradient characteristic coefficient based on the temperature rise gradient curve of the composite insulator axis is constructed as a new feature quantity for distinguishing decay-like insulators from non-decay-like insulators.

### 5.1. Presentation of Characteristic Coefficient

In high-humidity environments, when the temperature rise in a composite insulator is between 2 °C and 15 °C, and there is only one temperature rise area at the high-voltage end of the insulator, it is not possible to effectively diagnose a decay-like composite insulator by both the degree of temperature rise and the characteristics of the temperature rise area. As can be seen from the simulation results, the heat source of the decay-like composite insulator is mainly the internal mandrel, which is wrapped around the sheath, and the convective heat dissipation is impeded by the external sheath, while the heat is dissipated mainly by solid heat conduction. Thus, the temperature distribution is more even in the local area near the heat source. The heat source of the sheath-aged composite insulator is the part of the sheath surface with a high dielectric loss factor, and the surface of the sheath is in direct contact with the air, which facilitates convective heat dissipation; therefore, only the area near the heat source has a high temperature, and the temperature drops abruptly after moving away from the heat source.

In view of this, this section calculates the axial temperature rise gradient curve of the composite insulator based on the axial temperature rise distribution curve Δ*T* at the high-voltage end of the insulator by Equation (6), and the average variance of its temperature rise gradient is shown in Equation (7).

(5) $$\Delta T=[\Delta T_{1},\Delta T_{2},\ldots ,\Delta T_{i},\ldots ,\Delta T_{n}]$$

(6) $$\left\{\begin{array}{l} k_{i}=\frac{\Delta T_{i+1}-\Delta T_{i}}{l}\\ l=H/(n-1) \end{array}\right.i=0,1,2,\ldots ,n$$

where Δ*T_i_* is the temperature rise value of a single pixel point on the axial temperature rise curve of the composite insulator, *n* is the number of pixel points on the temperature rise curve, *H* is the insulation distance corresponding to the selected temperature rise curve on the composite insulator, and *l* is the distance between two pixel points on the temperature rise curve.

(7) $$\sigma =\sqrt{\frac{\sum_{i=1}^{n-1}{\left(k_{i}-\bar{k}\right)}^{2}}{n-1}}$$

(8) $$\bar{k}=\frac{\sum_{i=1}^{n}k_{i}}{n}$$

Let the maximum value of the temperature rise gradient be *k_max_* and define the product of the maximum value of the temperature rise gradient and the average variance of the temperature rise gradient as the temperature rise gradient characteristic coefficient *C_g_*, as shown in Equation (9), which characterises the rate of temperature change in the region of the axis on which the temperature rise curve lies and the degree of dispersion of the rate of change.

(9) $$C_{g}=\sigma k_{\max}$$

**Figure 17:**
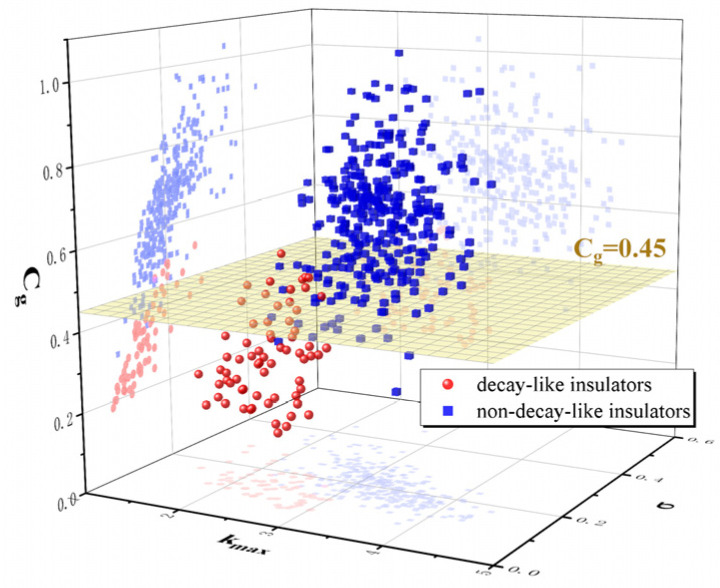
Statistics of characteristic coefficient of temperature rise gradient.

Statistical analysis of the temperature rise gradient characteristic coefficients (shown in Figure 17) of the 401 infrared images of the abnormally heated composite insulators at the high-voltage end obtained through field inspection and laboratory tests shows that 98.5% of the decay-like composite insulators have a temperature rise gradient characteristic coefficient of 0.45 or less, and only 3.7% of the non-decay-like composite insulators have a temperature rise gradient characteristic coefficient of 0.45 or less.

### 5.2. Application

**Figure 18:**
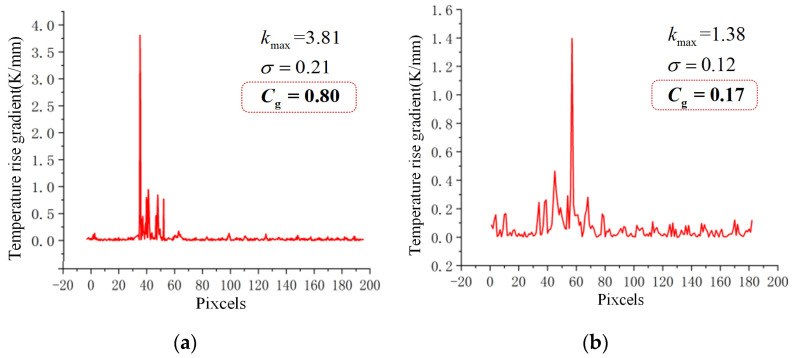
Temperature rise gradient curves of S1 and S2. (**a**) Axis temperature rise gradient curve of S1; (**b**) axis temperature rise gradient curve of S2.

The infrared image of the two composite insulator specimens (S1, S2) from Section 2 of this paper was processed according to the standard DL/T664-2008 and Equations (6) and (7) [27], to obtain the temperature rise gradient curve at the axis of the composite insulator, as shown in Figure 18.

As can be seen in Figure 18, the S1 composite insulator has a large rate of change in temperature rise, and the area with a large temperature rise gradient is more concentrated, with almost no change in temperature rise outside the area. The rate of change of temperature rise at the axis of the S2 composite insulator is smaller, and the temperature rise gradient is less discrete, which is in accordance with the thermal transfer characteristics of sheath ageing and decay-like composite insulators. Furthermore, the characteristic coefficient of the temperature rise gradient of the S1 composite insulator is greater than 0.45, while that of the S2 composite insulator is less than 0.45. Based on the criteria proposed in this paper, it can be preliminarily diagnosed that the S2 composite insulator is a decay-like composite insulator. The above tests further demonstrate that the characteristic coefficient of temperature rise gradient is an effective characteristic parameter to distinguish the presence of decay deterioration in composite insulators.

## 6. Conclusions

This paper presented a statistical analysis of the infrared temperature rise data of composite insulators collected in the field and from tests and showed that there is a blind spot in the diagnosis of decay-like insulators based only on two characteristic quantities, the temperature rise area and the temperature rise amplitude. Then, the heating mechanism and temperature distribution characteristics of composite insulators under the conditions of brittle defects and sheath ageing were studied, and a new infrared diagnostic feature, the temperature rise gradient coefficient, was proposed to identify abnormal heat sources. Its effectiveness was verified through experiments. The main conclusions of this paper are as follows.

(1) It is not possible to effectively diagnose a decay-like insulator with an abnormal temperature rise at the high-voltage end only, and with a temperature rise between 2 °C and 15 °C, by using temperature rise area and temperature rise amplitude information. Further screening is required with the help of new information on valid temperature rise characteristics.

(2) Due to the combined effects of different heat source locations and the different thermal conductivity of materials, under similar temperature rise amplitudes, the surface temperature rise distribution of sheath-aged insulators is more concentrated, and the surface temperature distribution of mandrel-deteriorated insulators is more even. The maximum temperature rise gradient *k_max_* and the average variance of the temperature rise gradient *σ* on the surface of the high-voltage end of the composite insulators with ageing sheaths and deteriorated mandrels differ significantly: at a temperature rise amplitude Δ*T* of around 10 °C, the maximum temperature rise gradient on the surface of the mandrel-deteriorated insulators is 1.546 °C/mm, while the maximum temperature rise gradient on the surface of the sheath-deteriorated insulators reaches 3.699 °C/mm. In addition, the average variance of the temperature rise gradient σ on the mandrel-deteriorated insulators is 0.2266, while the average variance of the temperature rise gradient σ on the sheath-deteriorated insulators reaches 0.4091. This provides an idea for proposing new infrared diagnostic features.

(3) In high-humidity environments, the temperature gradient characteristic coefficient *C_g_* of infrared images of composite insulators with abnormal heating at the high-voltage end is an effective method for diagnosing decay-like composite insulators. The accuracy rate of using the temperature gradient characteristic coefficient to diagnose brittle composite insulators is approximately 96%.

## Figures and Tables

**Table 1:** Electrical parameters of the composite insulator.

Specimen No.	Sheathing of Heat-Generating Area		Mandrel in the Heat-Generating Area	
	*ε*	tan*δ*	*ε*	tan*δ*
S1	7.05	0.158	5.97	0.023
S2	6.97	0.132	14.79	1.43

**Table 2:** FXBW-500/300 composite insulator parameters.

Model	Structure Height (mm)	Number of Umbrellas (Large/Small)	Mandrel Diameter (mm)
FXBW-500/300	5410	61(31/30)	36

**Table 3:** Thermodynamic parameters of composite insulator.

Material	Thermal Conductivity (W/(m·K))	Specific Thermal Capacity (J/(kg·K))	Density (kg/m^3^)
Silicone rubber	0.27	1700	1100
Mandrel	0.4	540	2000
Air (75% humidity)	0.0011	1.01	25

**Table 4:** Composite insulator temperature rise characteristic information.

Defect Type	Max. Surface Temperature Rise (°C)	Temperature Rise Area
Mandrel deterioration	11.4	High-voltage end fittings to between the second and third umbrella
Sheath ageing	10.3	

## Data Availability

The data presented in this study are available on request from the first authors and corresponding author.
